# Phylogenetic and Genome Analysis of 17 Novel Senecavirus A Isolates in Guangdong Province, 2017

**DOI:** 10.3389/fvets.2018.00314

**Published:** 2018-12-14

**Authors:** Yuan Sun, Jian Cheng, Rui-Ting Wu, Zi-Xian Wu, Jun-Wei Chen, Ying Luo, Qing-Mei Xie, Jing-Yun Ma

**Affiliations:** ^1^Animal Production and Environment Control, College of Animal Science, South China Agricultural University, Guangzhou, China; ^2^Key Laboratory of Animal Health Aquaculture and Environmental Control, Guangzhou, China; ^3^Guangdong Wens Foodstuffs Group Co., Ltd., Guangdong, China

**Keywords:** Senecavirus A, genome, phylogenetic analysis, Guangdong province, China

## Abstract

Senecavirus A (SVA), an emerging RNA virus, is considered to be associated with porcine idiopathic vesicular disease (PIVD). From February to September 2017, 17 novel SVA strains were isolated from samples with the vesicular disease from Guangdong Province, China. Full-length genomes and individual genes of the 17 new SVA isolates were genetically and phylogentically analyzed. Results showed that complete genomes, VP1, 3C, and 3D genes of these 17 novel SVA isolates revealed 96.5–99.8%, 95.1–99.9%, 95.6–100%, and 96.9–99.7% nucleotides identities, respectively. Phylogenetic analyses based on sequences of full-length genomes, VP1, 3C, and 3D genes indicated that 17 novel SVA isolates separated to three well-defined groups. Meanwhile, phylogenetic analysis for all available Chinese SVA strains demonstrated that 45 Chinese SVA strains clustered into five distinct groups with no significant relationship between strains from different provinces and/or years, including a newly emerging branch in China. This is the first comprehensive study of phylogenetic analysis for all available Chinese SVA strains, indicating the appearance of a new type of SVA strains and the complicated circulations with at least five different types of SVA strains in pigs in China.

## Introduction

**Figure 1:**
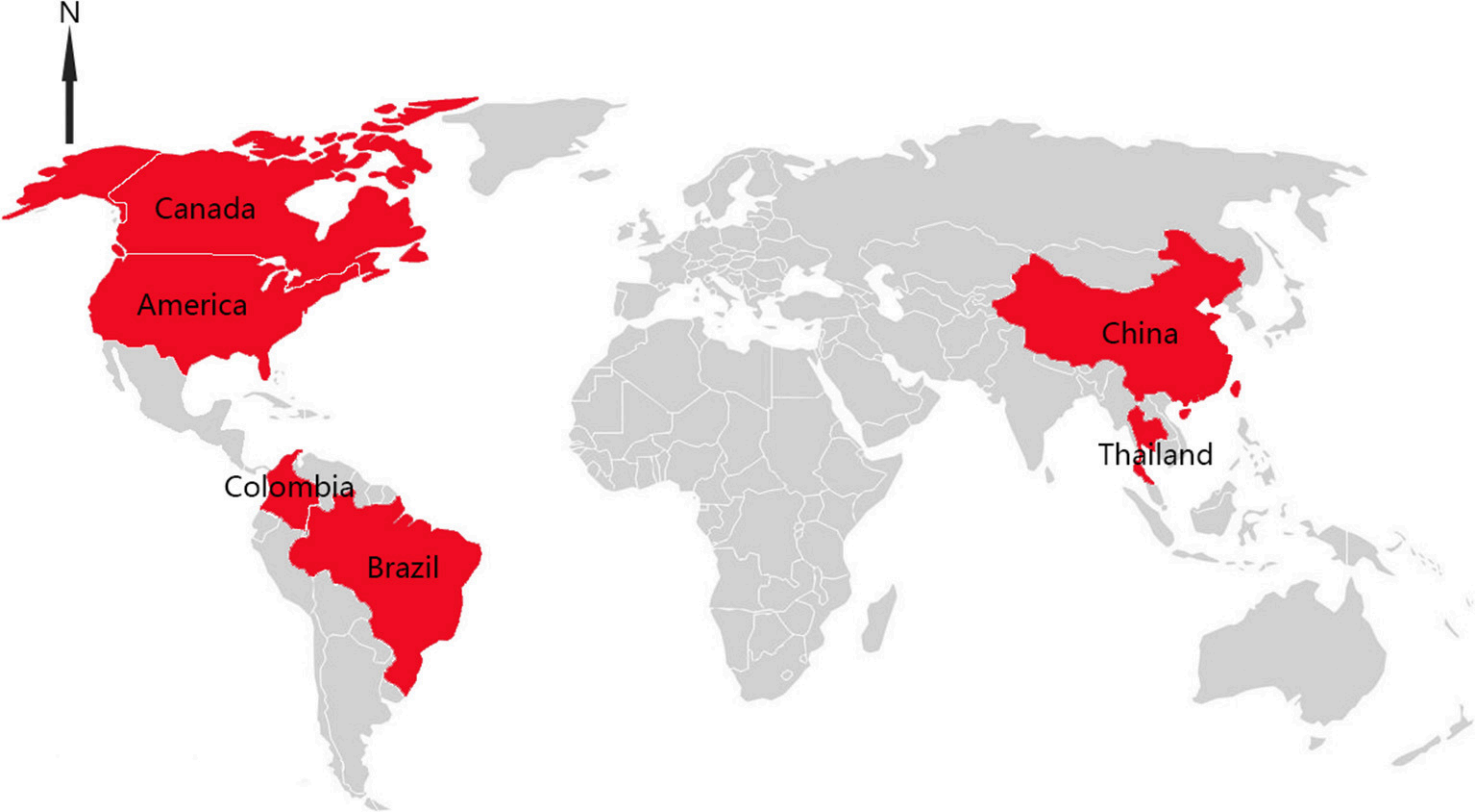
Distributions of SVA infection cases in the world.

Senecavirus A (SVA), first discovered as a cell contaminant in 2002, is a non-enveloped, single-stranded and positive-sense RNA virus. It belongs to the family *Piconaviridae* and is the only member of the genus *Senecavirus* (1, 2). The genome of SVA is approximately 7.2 kb in length and encodes four structural proteins (VP1 to VP4) and eight non-structural proteins (L, 2A, 2B, 2C, 3A, 3B, 3C, and 3D) (2). After large scale outbreaks of vesicular disease in sows as well as sudden neonatal death loss in Brazil which started at late 2014, SVA is identified to be the etiological agent of porcine idiopathic vesicular disease (PIVD) (3, 4). As a new causative agent, SVA has spread quickly and its clinical signs are difficult to be distinguished with infections of other viruses, including food-and-mouth disease virus (FMDV), swine vesicular disease virus (SVDV), vesicular exanthema of swine virus (VESV) and vesicular stomatitis virus (VSV), which have resulted in significant economic losses (5, 6). So far, there are six countries in Asian and American continents that have documented SVA associated with the vesicular disease in pigs (7–12) (Figure 1).

In China, the first SVA strain was isolated from pigs with classical symptoms of PIVD in Guangdong Province in 2015, and since then, increasing cases of SVA infections have emerged in more provinces, including Heilongjiang, Hebei, Henan, Hubei, Anhui, Fujian, and Guangxi (13–17). At present, nearly 30 full-length genomes of SVA reported from China are available in Genbank (access date: 19 August, 2018). Here, we report 17 novel SVA strains isolated in Guangdong Province from February to September in 2017 that are genetically separated into three distinct groups, and give a deep insight of the phylogenetic relationship between all available Chinese SVA strains.

## Materials and Methods

### Ethics Approval and Consent to Participate

This study was carried out in accordance with the recommendations of National Standards for Laboratory Animals of the People's Republic of China (GB149258-2010). The protocol was approved by Animal Research Committees of South China Agricultural University. Pigs used for the study were handled in accordance with good animal practices required by the Animal Ethics Procedures and Guidelines of the People's Republic of China.

### Sample Collection and Virus Detection

Seventeen vesicular and tissue samples of sows and piglets associated with vesicular disease were collected from 16 pig farms in five cities in Guangdong Province between February to September 2017 (Table 1). Viral RNA was extracted from tissue homogenates by using AxyPrep Body Fluid Viral DNA/RNA Miniprep Kit (Axygen, United States) according to the manufacturer's instructions. All RNA samples were stored at −80°C. SVA was detected by RT-PCR with primers described by Wu et al. (15).

**Table 1:** Details of 17 new SVA strains isolated in Guangdong Province, China.

**Name of sample**	**Sampling date**	**Sampling location**	**Sample**	**Pig group**
SVA/CHN/01/2017	27-Jun-17	Farm 1, Yangjiang	Viscera, lymph node	Sow
SVA/CHN/02/2017	25-Jul-17	Farm 2, Yangjiang	Vesicular fluid	Sow
SVA/CHN/03/2017	7-Jul-17	Farm 3, Zhaoqing	Vesicular fluid	Sow
SVA/CHN/04/2017	25-Jun-17	Farm 4, Qingyuan	Vesicular fluid	Sow
SVA/CHN/05/2017	2-Sep-17	Farm 5, Qingyuan	Vesicular fluid	Sow
SVA/CHN/06/2017	1-Sep-17	Farm 5, Qingyuan	Vesicular fluid	Sow
SVA/CHN/07/2017	19-Jul-17	Farm 6, Foshan	Lung, tongue, lymph node	Sow
SVA/CHN/08/2017	15-Jun-17	Farm7, Shaoguan	Vesicular fluid	Sow
SVA/CHN/09/2017	29-Aug-17	Farm 8, Shaoguan	Vesicular fluid	Sow
SVA/CHN/10/2017	3-May-17	Farm 9, Qingyuan	Vesicular fluid	Sow
SVA/CHN/11/2017	3-May-17	Farm 10, Zhaoqing	Vesicular fluid	Sow
SVA/CHN/12/2017	26-Apr-17	Farm 11, Qingyuan	Lung, lymph node	Piglet
SVA/CHN/13/2017	27-Apr-17	Farm 12, Qingyuan	Hoof	Sow
SVA/CHN/14/2017	26-Apr-17	Farm 13, Qingyuan	Hoof	Sow
SVA/CHN/15/2017	19-Apr-17	Farm 14, Qingyuan	Hoof, Viscera	Sow
SVA/CHN/16/2017	7-Jul-17	Farm 15, Qingyuan	Vesicular fluid	Sow
SVA/CHN/17/2017	16-Feb-17	Farm 16, Shaoguan	Vesicular fluid	NA

*NA, not available*.

### Virus Isolation

PK-15 cells that were cultured in Dulbecco's modified Eagle's medium (DMEM) supplemented with 10% fetal bovine serum (Thermo Scientific) were employed to isolate SVA. The tissue homogenates or vesicular fluids with phosphate-buffered saline (PBS; 20% w/v) were centrifuged for 15 min at 10,000 × g; then filtered suspended samples were inoculated into a 25-cm^2^ flask containing PK-15 cells at 80% confluency. The inoculated cells were incubated at 37°C in 5% CO_2_ and observed daily for cytopathic effect (CPE). When CPE appeared in 70% of the cells, viruses were harvested and the presence of virus was examined by RT-PCR.

### Genome Amplification and Sequence Analysis

Seven pairs of primers were utilized for the entire genome sequencing as previously described by Wu et al. (15). RNA samples were reverse-transcribed into cDNA and amplified using a one-step RT-PCR kit (TaKaRa, Dalian, China). The RT-PCR assay was performed with the following cycling conditions: 50°C for 30 min and 94°C for 5 min, 35 cycles of 94°C for 30 s, 55°C for 30 s, 72°C for 30 s, and a final extension at 72°C for 10 min. PCR products were purified by a Gel Band Purification Kit (Omega Bio-Tek, United States) and then cloned into the PMD-19T vector (TaKaRa, Dalian, China) and transformed *E. coli* DH5α competent cells. The recombinant plasmids were sequenced by the Beijing Genomics Institute (Shenzhen, Guangdong, China) Genomic sequences were assembled and aligned using the DNASTAR program (DNAStar V7.1, Madison, WI, United States). Phylogenetic trees were constructed using the neighbor-joining method in MEGA 7.0 software with bootstrap analysis of 1,000 replicates. Percentages of replicate trees in which the associated taxa clustered are shown as nearby branches (18–20).

## Results

Seventeen new SVA genomic sequences studied in this work were submitted to GenBank with the following accession numbers MG765550 to MG765566. The length of the complete genome was 7,286 bp. Nucleotide identities between these 17 new SVA strains ranged from 96.5 to 99.8%, and all 46 Chinese SVA strains showed 95.7–100% nucleotide identities. Except sharing low sequence identities with the prototype SVA strain SVV-001 (93.5–94.1%) and the strain ATCC PTA-5343 (93.1–94%), these 17 new Chinese strains were 97.1–98.6% identical to other USA strains. When compared to full-length sequences of Colombian, Brazilian, Canadian and Thai, the new 17 SVA strains shared 97.1–98.3%, 96.9–97.9%, 95.3–97.4%, and 94.8–95.8% complete genomic identities, respectively. Sequence analyses for individual genes of 17 new SVA strains showed that of the 12 genes, VP1, 3C and 3D genes had relatively low identities each with 95.1–99.5%, 95.9–99.7%, and 97–99.7%, while other nine genes were more conserved possessing 99.5–100% nucleotide identities.

**Figure 2:**
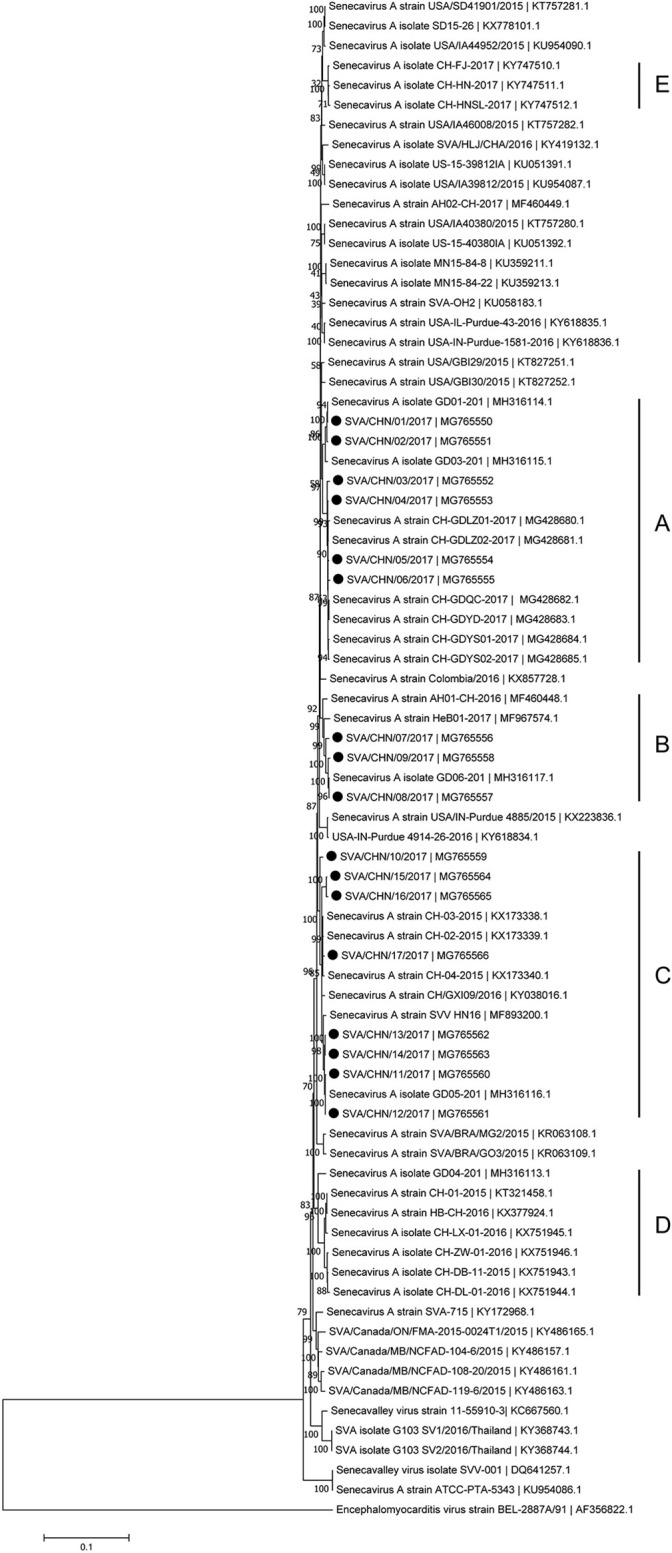
Phylogenetic relationships based on the complete genomes of 77 SVA strains identified from different countries and years. The tree was constructed using MEGA 7.0 software with neighbor-joining methods and 1,000 replicate sets on bootstrap analysis. 17 new complete genomes sequences studied in this work were indicated with “black blots”.

Phylogenetic analysis based on complete genomic sequences indicated that these 17 new SVA strains clustered into three distinct groups. The group A only contained sequences reported from Guangdong Province in 2017, including six new strains SVA/CHN/01/2017 to SVA/CHN/06/2017 and eight previously reported sequences. Meanwhile, the group A had a close relationship with two US strains identified in 2015. Fourteen SVA sequences in this group shared 97.7–100% identities with each other. Three new strains SVA/CHN/07/2017 to SVA/CHN/09/2017 and three other sequences that each were collected from Anhui, Hebei and Guangdong Province in 2016 and 2017 formed group B. Genomic sequences within this group shared 98.1–99.5% nucleotides identities. The remaining eight new strains and six previous Chinese sequences identified between 2015 and 2017 belonged to group C, and nucleotide identities in this group ranged from 97.2 to 99.9%. Besides these three groups, there were another two well-defined branches that only contained SVA strains in China. One group (D) included the first published Chinese SVA strain, CH-01-2015. Most of the strains in group D were reported in 2015 and 2016, but there was also a strain GD04-2017 (MH316113) which was identified in 2017. The last group (E) only included three sequences that were identified from Fujian and Henan provinces in 2017, and had a close relationship with three US strains identified in 2015. Nucleotide identities within and between each group were listed in Table 2. Compared with sequences in these five distinct groups, the remaining two Chinese strains, SVA/HLJ/CHA/2016 and AH02-CH-2017, separated from other Chinese SVA strains and each clustered with different US stains (Figure 2).

**Table 2:** Nucleotide identities within and between five distinct groups.

**Group name**	**A**	**B**	**C**	**D**	**E**
A	97.7–100%^*^	97–98.3%	96.5–97.5%	95.7–96.6%	97.3–97.9%
B		98.1–99.5%^*^	96.7–97.6%	96.2–97.7%	97.4–98.2%
C			97.2–99.9%^*^	95.9–97.6%	96.9–97.7%
D				97.7–100%^*^	96.2–96.8%
E					99.7–99.8%^*^

* *shows the nucleotide identities within each group*.

**Figure 3:**
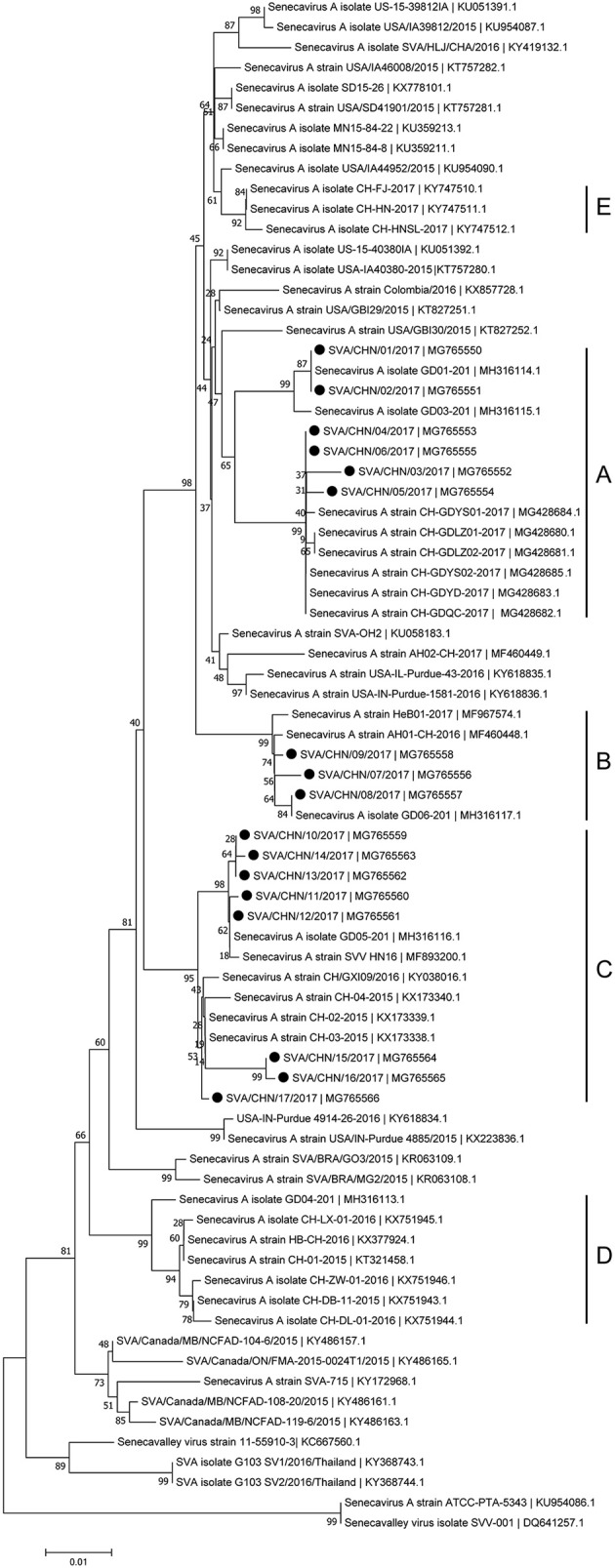
Phylogenetic relationships based on VP1 genes of SVA. The tree was constructed with the same method described previously. 17 new sequences of VP1 genes studied in this work were indicated with “black blots”.

**Figure 4:**
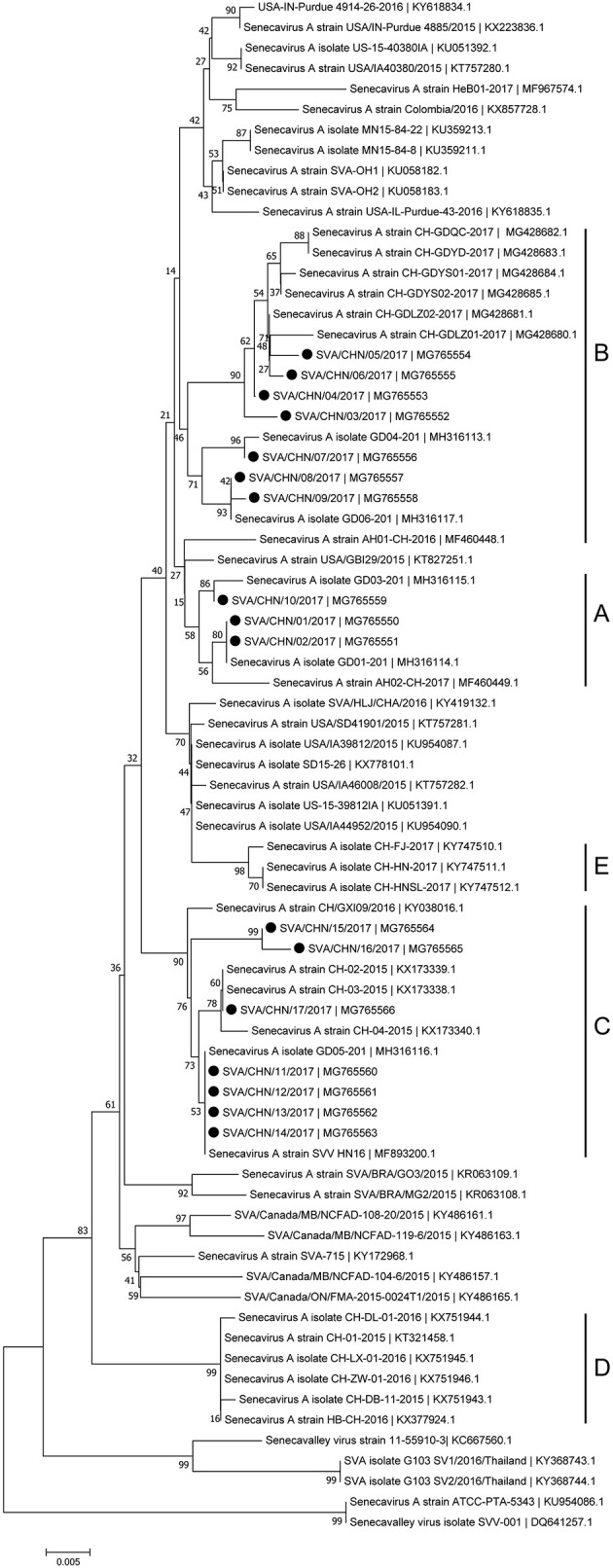
Phylogenetic relationships based on 3C genes of SVA. The tree was constructed with the same method described previously. 17 new sequences of 3C genes studied in this work were indicated with “black blots”.

**Figure 5:**
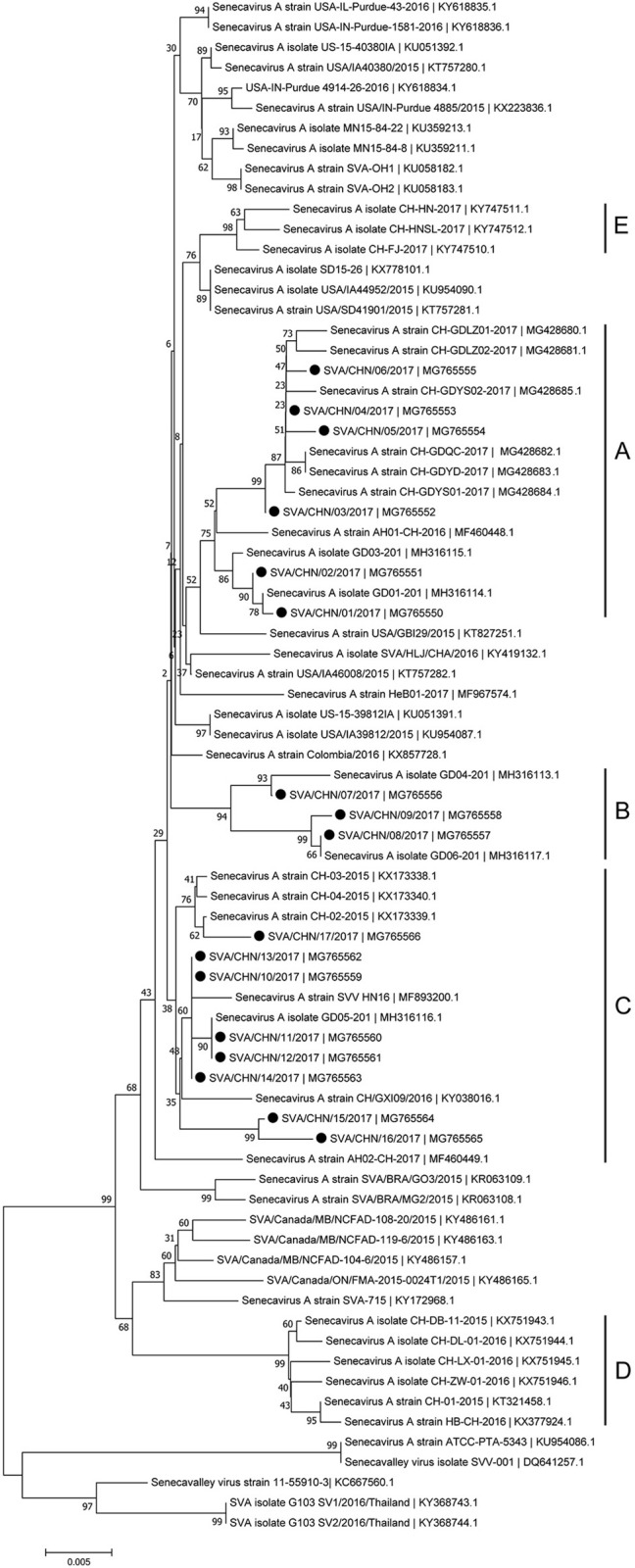
Phylogenetic relationships based on 3D genes of SVA. The tree was constructed with the same method described previously. 17 new sequences of 3D genes studied in this work were indicated with “black blots”.

Phylogenetic analyses based on sequences of VP1, 3C, and 3D genes also indicated the presence of five well-defined groups for Chinese SVA strains. For members in each group as described above, VP1 tree showed same results with the full-length genomic tree. The sequence GD04-2017 that belonged to group E based on VP1 genes and complete genomes was clustered into group B in the phylogenetic tree of 3D genes. This change also occurred in the phylogenetic tree of 3C genes. Besides this, in the tree of 3C genes four new SVA strains SVA/CHN/03/2017 to SVA/CHN/06/2017 and other five previous sequences were separated from other strains in group A and had a close relationship with strains in group B (Figures 3–5).

## Discussion

Since the first Chinese SVA infection case emerged in Guangdong Province on March 2015, 35 SVA genomic sequences including 17 new stains in this study has been reported from Guangdong Province which account for over 70% of Chinese isolates [(8, 12, 15); this paper]. Based on complete genomes and three individual genes of SVA, our results showed that these 17 new isolates and some other reported Chinese sequences clustered to three distinct groups with no significant relationship between strains from different provinces and/or years. Only a few of new SVA strains clustered with previous strains identified from Guangdong Province in 2017, while most of new strains clustered with sequences identified outside Guangdong Province in 2015–2017. 17 new SVA isolates in our study were determined from 16 pig farms, so the documented number of SVA infected farms in Guangdong Province has increased to 20 (12, 15). This indicates that since 2015 SVA infections have been rapidly expanding in Guangdong Province. Our results also showed that except one strain SVA/CHN/12/2017 collected from the piglet, most new strains were achieved from sows, which was consistent with findings of Zhang et al. (6) that SVA cases in 2017 in China were mainly identified in adult pigs.

Besides strains identified from Guangdong Province, there still are 10 SVA strains reported from other seven provinces in China. Combined our results and previous findings, by now there have been five different groups of SVA strains, which indicates different genotypes of SVA strains co-exist in China (8, 17). One group including three novel SVA strains SVA/CHN/07/2017 to SVA/CHN/06/2017 is a newly emerging group, implying the appearance of a new type of SVA strains and the complicated prevalence of SVA in pigs in China. Zhang et al. (6) reported that the recent Chinese SVA strains were closely related to current US strains. Our phylogenetic analyses based on 3C and 3D genes for all available Chinese strains showed that SVA strains identified in 2017 were all close to US strains identified in 2015, which is consist with Zhang et al.'s observations. However, the results based on complete genomes and VP1 genes indicated that a strain GD04-2017 were separated from other sequences identified in 2017 and clustered into a group which was close to Brazilian sequences and contained the first Chinese strain CH-01-2015. Three strains CH-01-2015, HB-CH-2016 and CH-LX-01-2016 in this group were employed in the phylogenetic analysis of Xu et al. (11) to investigate relationships between 33 genomes of SVA strains from US, Brazil, Canada, and China, and they obtained that these SVA sequences clustered in four groups according to different countries. However, according to our results there was no significant relationship between sequences from different countries, especially for SVA strains in China. Therefore, to better understand the diversity and evolutionary relationships of SVA strains, it's important to use more whole-genome sequences or variable individual genes sequences.

By now, five, seven and 33 genomes of SVA strains have been reported in China in 2015, 2016, and 2017, respectively. Continuous descriptions of SVA infections in China especially plenty of reports in 2017 suggests that SVA may spread across the country in the future. Better understanding spreading routes of SVA is crucial for its control, while information on SVA transmission remains sparse. In our investigations we have found that cohabitation and contamination of feed or pigs carriage contribute to the disseminations of SVA, which is consistent with previous findings (5). Although pigs are a natural host of SVA, this virus has been detected in mice and houseflies (21), the role of non-swine animals in SVA transmission requires further studies. Meanwhile, more efforts are warranted to focus on SVA monitoring and managements, e.g., rapid and specific diagnostics, isolations of infected animals and vaccination strategies.

## Conclusions

In conclusion, we reported 17 novel SVA isolates collected from Guangdong Province in 2017 and analyzed the phylogenetic relationships of all available Chinese SVA strains based on sequences of complete genomes, VP1, 3C, and 3D genes. Our results indicated the circulations of five different types of SVA strains in pigs in China, including a newly emerging type. Further studies based on more information of molecular epidemiology will help better understanding origin, evolution and transmission patterns of SVA in pigs in China.

## Author Contributions

J-YM conceived and designed the study, and critically revised the manuscript. YS conducted data analysis and wrote the manuscript. JC performed the experiments. R-TW helped in genomic data analyzed. Z-XW, J-WC, and YL helped in experimental implementation. Q-MX helped in study design. All authors read and approved the final manuscript.

### Conflict of Interest Statement

The authors declare that the research was conducted in the absence of any commercial or financial relationships that could be construed as a potential conflict of interest.
